# Triglyceride-glucose index threshold for cardiovascular mortality in hypertensive individuals - URRAH project

**DOI:** 10.1016/j.ajpc.2025.101053

**Published:** 2025-06-29

**Authors:** Lanfranco D’Elia, Ferruccio Galletti, Masulli Maria, Agostino Virdis, Edoardo Casiglia, Valerie Tikhonoff, Fabio Angeli, Carlo Maria Barbagallo, Michele Bombelli, Federica Cappelli, Rosario Cianci, Michele Ciccarelli, Arrigo F G Cicero, Massimo Cirillo, Pietro Cirillo, Giovambattista Desideri, Claudio Ferri, Loreto Gesualdo, Cristina Giannattasio, Guido Grassi, Guido Iaccarino, Luciano Lippa, Francesca Mallamaci, Alessandro Maloberti, Stefano Masi, Alberto Mazza, Alessandro Mengozzi, Maria Lorenza Muiesan, Pietro Nazzaro, Paolo Palatini, Gianfranco Parati, Roberto Pontremoli, Fosca Quarti-Trevano, Marcello Rattazzi, Gianpaolo Reboldi, Giulia Rivasi, Elisa Russo, Massimo Salvetti, Giuliano Tocci, Andrea Ungar, Paolo Verdecchia, Francesca Viazzi, Massimo Volpe, Claudio Borghi, Working Group on Uric Acid and Cardiovascular Risk of the Italian Society of Hypertension (SIIA)

**Affiliations:** a Department of Clinical Medicine and Surgery, "Federico II" University of Naples, 80131 Naples, Italy; b Department of Clinical and Experimental Medicine, University of Pisa, 56126 Pisa, Italy; c Studium Patavinum, Department of Medicine, University of Padua, 35100 Padua, Italy; d Department of Medicine, University of Padua, 35100 Padua, Italy; e Department of Medicine and Technological Innovation (DiMIT), University of Insubria, Varese, Italy; f Department of Medicine and Cardiopulmonary Rehabilitation, Maugeri Care and Research Institutes, IRCCS Tradate (VA), Italy; g Department of Health Promotion, Mother and Child Care, Internal Medicine and Medical Specialties (PROMISE), University of Palermo, 90100 Palermo, Italy; h Internal Medicine, Pio XI Hospital of Desio, ASST Brianza, Department of Medicine and Surgery, University of Milano, Bicocca, Italy; i Department of Translational and Precision Medicine, Sapienza University of Rome, 00185 Rome, Italy; j Department of Medicine and Surgery, University of Salerno, Salerno, Italy; k Hypertension and Cardiovascular Disease Research Center, Medical and Surgical Sciences Department, Alma Mater Studiorum University of Bologna, 40126 Bologna, Italy; l Cardiovascular medicine unit, Heart-Chest-Vascular Department, IRCCS Azienda Ospedaliero-Universitaria di Bologna, 40126 Bologna, Italy; m Dipartimento Scuola Medica Salernitana, Università di Salerno, Baronissi SA, Italy; n Dipartimento di Medicina di Precisione e Rigenerativa e Area Jonica (DiMePRe-J), "Aldo Moro" University of Bari, 70122 Bari, Italy; o Department of Clinical, Internal Medicine, Anesthesiologic and Cardiovascular Sciences, Sapienza University of Rome, 00161 Rome, Italy; p Department of Life, Health and Environmental Sciences, University of L'Aquila, 67100 L'Aquila, Italy; q Cardiology IV, "A.De Gasperi's" Department, Niguarda Ca' Granda Hospital, 20162 Milan, Italy; r School of Medicine and Surgery, Milano-Bicocca University, 20126 Milan, Italy; s Clinica Medica, Department of Medicine and Surgery, University of Milano-Bicocca, 20900 Monza, Italy; t Italian Society of General Medicine (SIMG), 67051 Avezzano, Italy; u Department of Nephrology, Dialysis and Transplantation GOM "Bianchi-Melacrino-Morelli" & CNR-IFC, Institute of Clinical Physiology, Research Unit of Clinical Epidemiology and Physiopathology of Renal Diseases and Hypertension (European Society of Hypertension, ESH, Excellence Centre) of Reggio Calabria, Italy; v Department of Internal Medicine, Santa Maria Della Misericordia General Hospital, AULSS 5 Polesana, 45100 Rovigo, Italy; w Center for Translational and Experimental Cardiology (CTEC), Department of Cardiology, University Hospital Zurich, University of Zurich, 8952 Schlieren, Switzerland; x Dipartimento Scuola Medica Salernitana, Università di Salerno, 84084 Baronissi-SA, Italy; y Department of Clinical and Experimental Sciences and "Centro studi diagnosi e cura dell'ipertensione arteriosa e del rischio cardiovascolare ", University of Brescia, 25121 Brescia, Italy; z Department of Precision and Regenerative Medicine and Jonic Area (DiMePRe-J), Neurosciences and Sense Organs, University of Bari Medical School, 70122 Bari, Italy; aa S.Luca Hospital, IRCCS, Istituto Auxologico Italiano, 20149 Milan, Italy; ab Department of Medicine and Surgery, University of Milano-Bicocca, 20126 Milan, Italy; ac Dipartimento di Medicina Interna e Specialità Mediche, Università degli Studi di Genova, Italy; ad IRCCS Ospedale Policlinico San Martino, Genova, Italy; ae Department of Medicine-DIMED, University of Padova, Medicina Interna 1°, Ca' Foncello University Hospital, 31100 Treviso, Italy; af Department of Medicine and Surgery, University of Perugia, 06100 Perugia, Italy; ag Department of Geriatric and Intensive Care Medicine, Careggi Hospital, University of Florence, 50121 Florence, Italy; ah Department of Clinical and Molecular Medicine, University of Rome Sapienza, Sant'Andrea Hospital, 00189 Rome, Italy; ai Hospital S. Maria della Misericordia, 06100 Perugia, Italy; aj Dipartimento di Medicina Clinica e Molecolare, Università di Roma Sapienza, Roma, Italy; ak IRCCS San Raffaele Roma, Italy

**Keywords:** Triglyceride-glucose index, Cardiovascular mortality, Hypertension, Insulin resistance

## Abstract

**Aims:**

The triglyceride-glucose (TyG) index is a surrogate marker of insulin resistance (IR). Data regarding this topic is constantly increasing, however, few and heterogeneous data are available on the relationship between this index and cardiovascular mortality risk in hypertensive populations. In this context, we aimed to explore the relationship between TyG and cardiovascular mortality in a large sample of hypertensive individuals from the URRAH cohort.

**Methods:**

A total of 12,275 hypertensive participants without previous cardiovascular events were included in this analysis. The risk of cardiovascular mortality was evaluated by the Cox regression analysis and competing risk regression analysis.

**Results:**

During a median follow-up of 10.5 years, 2151 deaths occurred, of which 986 were from cardiovascular disease. A linear association between TyG and cardiovascular mortality was found, in particular for a 1-standard deviation increase in TyG there was a significantly increased risk of 33 % (*p* < 0.0001). Furthermore, after stratification by the optimal cut-off point (4.54 Units), participants with TyG above the cut-off had a significantly increased risk of 67 % of cardiovascular mortality when compared with those with TyG below the cut-off (*p* < 0.0001). These results were also confirmed after adjustment for potential confounders.

**Conclusions:**

The results of this study indicate that this TyG threshold is predictive of an increased risk of cardiovascular mortality in a large sample of hypertensive individuals. This cut-off can identify individuals at higher mortality risk in already high-risk patients, with a low-cost and simple non-invasive marker.

## Graphical abstract

**Figure:**
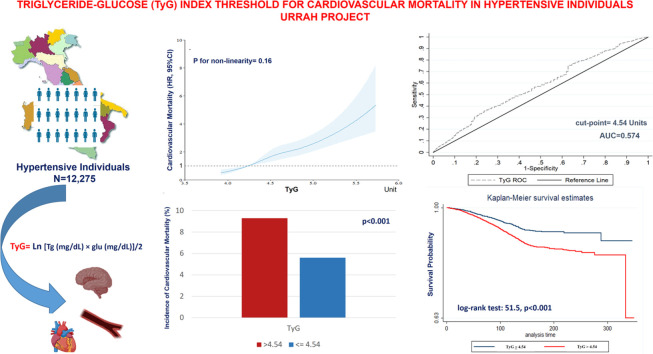

## 1 Introduction

Hypertension is one of the main modifiable risk factors for cardiovascular diseases that affects more than one billion people worldwide [1], with its predicted worldwide increase of at least 30 % by 2025 [2]. Hypertension can occur together with other cardio-metabolic conditions, such as insulin resistance (IR) [3,4], in turn, associated with an increased risk of cardiovascular disease [5]. Therefore, an early identification of IR is important to classify individuals at particularly high cardiovascular risk. Several methods have been tested to assess IR in clinical practice and epidemiological studies, but all these tools require insulin level measurement, hence a limitation because insulin determination is not routinely performed and is relatively costly [6].

Nonetheless, to overcome the limitations of insulin-based methods, a novel surrogate marker - the triglyceride-glucose (TyG) index - was introduced in 2008 as a simple and low-cost alternative that does not require insulin measurement [7,8]. Although the TyG index was originally proposed over a decade ago, its application in cardiovascular epidemiology as a reliable indicator of IR has significantly increased only in recent years, with a growing body of evidence supporting its prognostic value in diverse clinical and population settings [7,[9], [10], [11]]. It has a high correlation with hyperinsulinemic-euglycemic clamp (gold-standard measurement of IR) [8] and it is a better expression of IR than HOMA-IR in some settings [9,10]. Observational studies showed a substantial positive relationship between TyG and mortality risk in the general population [7,[11], [12], [13]] and cardiovascular disease [7,14], likewise with cardiovascular risk factors in different settings [7,10,15,16]. More recently, TyG thresholds to define mortality risk have been proposed in the general population [13]. However, published data on the predictive role of TyG on mortality in hypertensive people are few and heterogeneous, and no TyG threshold was detected [[17], [18], [19]].

In consideration of the huge prevalence and future incidence of hypertension worldwide, the crucial role of IR in cardiovascular risk stratification, the strong predictive role of TyG, and its threshold absence to identify mortality risk in hypertensive people, we aimed to evaluate the association of TyG index with mortality in the hypertensive URRAH (URic acid Right for heArt Health) population [20], to identify the best TyG threshold predictive for cardiovascular mortality.

## 2 Methods

### 2.1 Study population

The URRAH database is a multicenter retrospective, observational cohort study, which involves data from several cohorts recruited within Italian hypertension centers and distributed in almost all the Italian regions (age: 18–95 years). More details of the URRAH project have been published previously [21,22]. This article was written according to the Strengthening the Reporting of Observational Studies in Epidemiology (STROBE) statement [23] (Supplemental Table 1). For the present study, 12,275 participants with hypertension were considered, after the exclusion of participants without hypertension, or without a complete database, participants with triglyceride levels >400 mg/dL or blood glucose <60 or >300 mg/dl to avoid distortion of the statistical distribution and clinical interpretation of the TyG index due to extreme values, and individuals with previous cardiovascular events were excluded to reduce confounding from secondary prevention therapies and to focus the analysis on primary prevention, ensuring a more homogeneous hypertensive population for risk stratification (total of excluded, *n* = 14,803). The included participants were older than the excluded participants, and as expected, they had a substantially worse anthropometric, metabolic, and hemodynamic profile, and higher TyG (Supplemental Table 2).

### 2.2 Data collection

The URRAH study procedures have been extensively described [21,22]. In particular, hypertension was defined as systolic blood pressure (BP) ≥ 140 or diastolic BP ≥90 mmHg or current antihypertensive drug treatment [24]. Body weight and height were measured on a standard beam balance scale with an attached ruler. Body mass index (BMI) was measured according to the formula weight (kg)/height^2^ (m), in particular, overweight was defined as a BMI between 25 and 29.9 kg/m^2^, and obesity as a BMI ≥ 29.9 kg/m^2^. The estimated glomerular filtration rate (eGFR) was calculated using the Chronic Kidney Disease Epidemiology Collaboration (CKD-EPI) 2009 equation [25]. The overt kidney dysfunction was defined as eGFR equal-below 60 ml/min per 1.73 m^2^ [26]. Low-density lipoprotein (LDL) cholesterol was calculated by the Friedewald equation: total cholesterol – (triglycerides/5 + HDL cholesterol). Type 2 diabetes mellitus (DM) was defined according to standardized criteria (treatment with antidiabetic drugs, fasting plasma glucose ≥126 mg/ dL, or hemoglobin A1c ≥ 48 mmol/mol at baseline examination). High serum uric acid (SUA) levels was defined based on the previously described URRAH cut-off levels (i.e. SUA >5.6 mg/dL) [21]. Therapy for high triglycerides was considered with the use of fibrates, niacin , and fish oil, while statins were considered separately. The TyG index was calculated using the following formula: Ln (TG [mg/dL] * fasting glucose [mg/dL])/2 [7,8].

### 2.3 Outcome

All-cause and cardiovascular mortality were evaluated at the end of the follow-up. Information on patients who had died was obtained from hospital records or death certificates. Mortality from cardiovascular disease was coded according to the International Classification of Diseases, Tenth Revision – ICD10 (i.e., myocardial infarction, heart failure, angina pectoris, transient ischemic attack, stroke, and hypertensive complications) [21].

### 2.4 Statistical analysis

The statistical analyses were performed using the SPSS software (version 29 - SPSS Inc., Chicago, Il), the statistical package R (The R Project for Statistical Computing, version 4.3.1), and STATA Corp. software (version 13).

Because eGFR, SUA, total cholesterol, HDL cholesterol, triglycerides, glucose, heart rate, and LDL cholesterol were non-normal distributed (the Kolmogorov–Smirnov test, *p* < 0.01), log-transformed values were used in the analyses. The analysis of variance for continuous data and the chi-squared test to evaluate differences between categorical variables were used to assess statistical differences between groups’ characteristics. Bivariate relationships between the variables under investigation were evaluated by Pearson’s correlation analysis. To assess the type of association between TyG and cardiovascular mortality, restricted cubic splines (RCS) regression models with 4 knots (5th-reference, 35th, 65th, and 95th percentiles) were utilized. Given the linear association between TyG and cardiovascular mortality, a Cox regression analysis was used to preliminary assess the predictive power of the index. Furthermore, the receiver operative characteristic (ROC) curve for time-dependent outcome was carried out to identify the optimal cut-point (Youden’s criterion) of the association of continuous baseline TyG with cardiovascular mortality at follow-up (cut-point=4.54 Units). According to this cut-point, the sample was stratified into two groups and analyzed. To analyze the predictive role of baseline TyG (categorical data) on the risk of cardiovascular mortality, Kaplan–Meier survival curves, log-rank tests, Cox proportional-hazards multivariate models, and competing risk analysis were used. The impact of potential confounding factors with biological plausibility was explored using multivariate models adjusted for baseline age, sex, BMI, DM, BP, cigarette smoking, statins use, antihypertensive therapy, serum uric acid, renal function, therapy for high triglycerides, total cholesterol, HDL cholesterol, heart rate, and LDL cholesterol (instead of total cholesterol and HDL cholesterol). The proportional hazard (PH) assumption was assessed by the visual inspection of Kaplan-Meyer curves and testing of Schoenfeld residuals. The Akaike information criteria (AIC, lower is better) and Bayesian Information Criterion (BIC, lower is better) were used to assess the relative goodness of fit. Harrell's c-index (higher is better) was used to assess the discriminatory power of the independent variables.

Subgroup analyses were carried out to identify potential factors, among participants’ characteristics, that may affect the predictive role of TyG on cardiovascular mortality (i.e. sex: males, females; age: ≥70 years, <70 years; DM: yes, no; smokers: yes, no; LDL cholesterol: ≥130 mg/dl, <130 mg/dl; systolic BP: ≥140 mmHg, <140 mmHg; renal function: eGFR ≤60 ml/min/1.73 m^2^, >60 ml/min/1.73 m^2^; high SUA: yes - ≥5.6 mg/dl, No - <5.6 mg/dl); body weight: normal - BMI <25 kg/m^2^, overweight - BMI 25–29.9 kg/m^2^, obesity - BMI ≥ 30 kg/m^2^.

The results are reported as mean (or geometric mean) with standard deviation (SD), percentages, or hazard ratio (HR), or sub-hazard ratio (SHR) and 95 %CI (95 % confidence interval), unless otherwise indicated. Two-sided P values below 0.05 were considered statistically significant.

## 3 Results

### 3.1 Characteristics of the sample

At baseline, the mean age of the whole sample of hypertensive participants was 59.5 years; 49.7 % were men, 45.3 % of the participants were overweight and 20.4 % were obese, 38.9 % were on regular antihypertensive treatment, 11.8 % were diabetic, and 21.7 % were smokers (Table 1). A total of 6.0 % of the participants were on treatment with statins and about 1 % were on treatment with therapy for high triglycerides. The baseline TyG average was 4.65 (median: 4.64, SD: 0.27).

**Table 1:** Baseline characteristics of all the study participants and stratified according to TyG threshold predictive for cardiovascular mortality.

**Variables**	**Total sample**	**TyG >4.54 Units**	**TyG ≤4.54 Units**
No. of participants (%)	12,275	8029 (65.4)	4246 (34.6)
Sex (M/F - %)	49.7/50.3	49.7/50.3	49.7/50.3
Age (yrs)	59.5 (14.3)	60.5 (13.8)*	57.7 (14.9)
Age >70 years (yes - %)	28.7	30.5*	25.3
BMI (kg/m^2^)	26.9 (4.2)	27.6 (4.2)*	25.6 (4.0)
Normal-weight (%)	34.3	27.0*	48.1
Overweight (%)	45.3	48.7*	38.7
Obesity (%)	20.4	24.3*	13.2
Systolic BP (mmHg)	154.9 (20.6)	156.3 (21.0)*	152.2 (19.6)
Systolic BP >140 mmHg (yes - %)	81.9	83.3 *	79.3
Diastolic BP (mmHg)	91.5 (11.5)	92.0 (11.6)*	90.5 (11.2)
Heart rate (bpm)^1^^,^^3^	72.4 (1.2)	72.4 (1.2)*	71.6 (1.2)
eGFR (mL/min/1.73 m^2^) 1	77.6 (1.4)	75.5 (1.4)*	83.2 (1.4)
Kidney dysfunction (yes - %)	17.2	19.9*	12.2
Glucose (mg/dl) 1	97.7 (1.2)	102.3 (1.2)*	90.7 (1.1)
Type 2 Diabetes Mellitus (yes - %)	11.8	15.9*	4.0
Total Cholesterol (mg/dl) 1^,^^2^	208.9 (1.2)	218.8 (1.2)*	195.6 (1.2)
LDL cholesterol (mg/dl) 1^,^^3^	128.8 (1.3)	134.9 (1.3)*	120.2 (1.3)
LDL cholesterol >130 mg/dl (yes - %)	54.7	60.5*	44.2
HDL cholesterol (mg/dl) 1^,^^3^	51.3 (1.3)	48.9 (1.3)*	56.3 (1.3)
Triglycerides (mg/dl) 1	112.2 (1.6)	144.5 (1.4)*	69.2 (1.3)
Serum Uric Acid (mg/dl) 1	5.0 (1.3)	5.2 (1.3)*	4.5 (1.3)
High Serum Uric acid (yes - %)	36.8	43.3*	24.6
Therapy for high triglycerides (yes - %)	1.1	1.5*	0.2
Antihypertensive therapy (yes - %)	38.9	41.5*	34.0
Statin use (%)	6.0	6.3*	5.3
Cigarette Smoking (%)	21.7	22.8*	19.6
TyG (Unit)	4.65 (0.27)	4.80 (0.19)*	4.37 (0.13)

BMI: body mass index; BP: Blood Pressure; eGFR: estimated glomerular filtration rate; Normal-weight: BMI <25 kg/m^2^, Overweight: BMI 25–29.9 kg/m^2^, obesity: BMI ≥ 30 kg/m^2^; TyG: triglyceride-glucose index.

1 Data are expressed as geometric mean (SD);.

2 sample reduced by 5 %;.

3 sample reduced by 15 %;.

⁎ >4.54 vs ≤4.54 Units: *p* < 0.05;.

### 3.2 TyG and cardiovascular risk factors

The analysis of the correlation between TyG and the most relevant baseline characteristics of participants showed a significant association with age (*r* = 0.09), BMI (*r* = 0.29), systolic (*r* = 0.10), and diastolic BP (*r* = 0.06), lipid profile (total cholesterol: *r* = 0.30, HDL cholesterol: *r*= −0.32, LDL cholesterol: *r* = 0.18), SUA (*r* = 0.29), and eGFR (*r*= −0.18).

### 3.3 TyG and cardiovascular mortality

During a median follow-up of 10.5 years (126 months, 25th-75th: 64–181 months), 2151 (17.5 %) all-cause deaths occurred, 986 (45.8 %) of which were due to cardiovascular causes. At baseline, those who died from cardiovascular disease were older, with higher TyG, systolic BP, SUA, heart rate, and prevalence of DM and smoking habit, and lower renal function, prevalence of male participants, antihypertensive therapy, use of statins and treatment for high triglycerides (Supplemental Table 3).

**Fig. 1:**
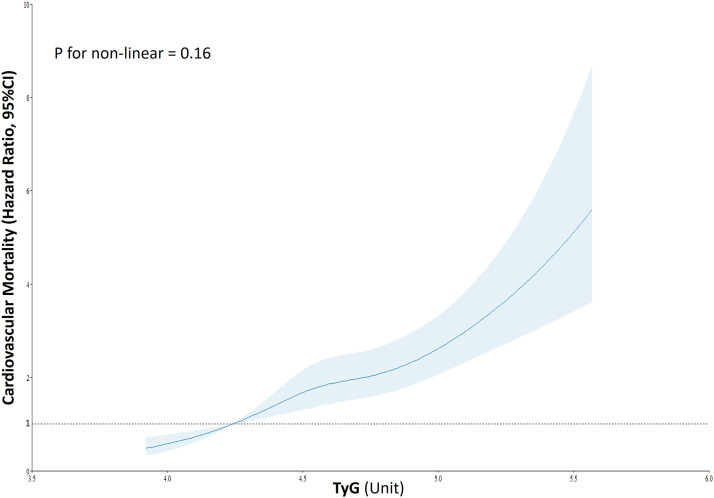
Association between the triglyceride-glucose index (TyG) and risk of cardiovascular mortality using a restricted cubic spline regression model. Solid lines indicate hazard ratios (HRs), and shadow shapes indicate 95 % confidence intervals (CIs).

RCS regression model detected a positive linear relationship between TyG and cardiovascular mortality (test for overall: *p* < 0.001, test for non-linearity: *p* = 0.16) (Fig. 1). A preliminary Cox regression analysis showed that for a 1-SD increase in TyG, HR was 1.35 (95 %CI: 1.27–1.43, *p* < 0.0001). This association was confirmed upon adjustment for the main confounders (HR: 1.16, 95 %CI: 1.07–1.26, *p* < 0.0001), as well as in the model with HDL cholesterol and heart rate as covariates (HR: 1.14, 95 %CI: 1.04–1.24, *p* = 0.006). Similar results were detected in the competing risk analysis, in the unadjusted model (for 1-SD increase in TyG, SHR: 1.33, 95 %CI: 1.25–1.42, *p* < 0.0001), in the model adjusted for the main confounders (SHR: 1.14, 95 %CI: 1.05–1.23, *p* = 0.001), and in the model also adjusting for HDL cholesterol and heart rate (SHR: 1.10, 95 %CI: 1.01–1.21, *p* = 0.03).

### 3.4 TyG threshold for cardiovascular mortality

**Fig. 2:**
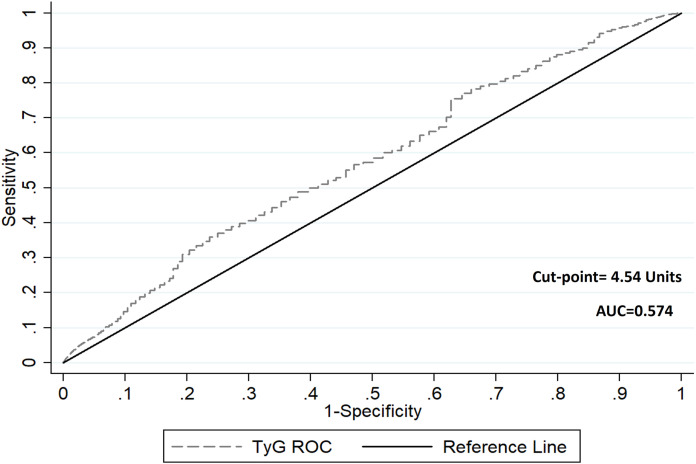
Time-dependent receiver-operating characteristic ROC curve for the relationship between triglyceride-glucose (TyG) index and cardiovascular mortality.

Next, the time-dependent ROC curve for the relationship between TyG and cardiovascular mortality was created (AUC: 0.574) (Fig. 2). Given the stratification based on the optimal cut-point by the ROC curve (cut-point= 4.54 Units; sensitivity: 78 %, specificity: 37 %), we evaluated the predictive role of TyG for cardiovascular mortality.

The TyG group with a value >4.54 Units had higher baseline age, BMI, BP, heart rate, a worse metabolic profile, and lower renal function than those with TyG lower than 4.54 Units (Table 1).

**Fig. 3:**
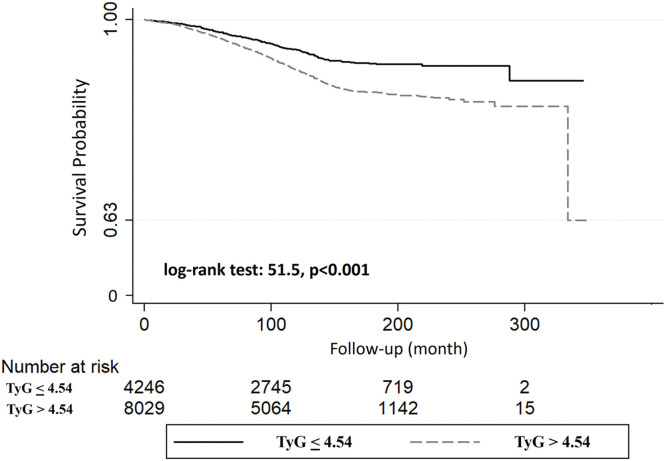
Kaplan-Meier curve for cardiovascular mortality for people with triglyceride-glucose (TyG) index lower and above 4.54 Units.

Participants with TyG >4.54 Units had a higher incidence of cardiovascular mortality than participants with TyG below 4.54 Units (9.3 % vs 5.6 %, *p* < 0.001). The Kaplan-Meier curve for cardiovascular mortality is shown in Fig. 3. In particular, participants with values above 4.54 Unit had a significantly higher probability of cardiovascular mortality than those with TyG below 4.54 Units (log-rank test: 51.5, *p* < 0.001). Cox-regression analysis confirmed the predictive role of the cut-point on cardiovascular mortality, which showed a greater risk of mortality in participants with TyG above than below 4.54 Units (HR: 1.69, 95 %CI: 1.46–1.96, *p* < 0.001). This predictive role was also detected after adjustment for main potential confounders (HR: 1.31, 95 %CI: 1.11–1.55, *p* = 0.001), after adjustment for HDL cholesterol and heart rate (HR: 1.22, 95 %CI: 1.03–1.46, *p* = 0.02), or after adjustment for LDL cholesterol instead of total cholesterol and HDL cholesterol (HR: 1.20, 95 %CI: 1.01–1.42, *p* = 0.03). A violation of the PH assumption was not detected through visual inspection of Kaplan-Meyer curves and a test based on Schoenfeld residuals (*p* = 0.53).

Moreover, the AIC and BIC indicated that including TyG improved the model fit compared to not including it (delta AIC: −10 and BIC: −3).

A similar trend was confirmed in the competing risk analysis, in particular, participants with TyG above versus below 4.54 Units had a significantly higher risk both in unadjusted (SHR: 1.67, 95 %CI: 1.44–1.93) and adjusted models (Table 2).

**Table 2:** Competing risk analysis on cardiovascular mortality according to the threshold of TyG index (*n* = 12,275).

	**TyG > 4.54 Units vs TyG ≤ 4.54 Units**	**p-value**
	**SHR (95 % CI)**	
Unadjusted	1.67 (1.44–1.93)	<0.0001
Multivariable Model 1^a^	1.20 (1.02–1.40)	0.02
Multivariable Model 2^b^	1.29 (1.10–1.52)	0.002
Multivariable Model 3^c^	1.20 (1.01–1.43)	0.03
Multivariable Model 4^d^	1.19 (1.01–1.41)	0.04

BMI: body mass index; CI: confidence interval; eGFR: estimated glomerular filtration rate; SHR: sub-hazard ratio; TyG: triglyceride-glucose index.

a Model 1 adjusted for baseline age, sex, BMI, systolic blood pressure, statins use, serum uric acid, type 2 diabetes mellitus, eGFR, cigarette smoking, therapy for high triglycerides, therapy for hypertension;.

b Model 2 adjusted for Model 1 plus baseline total cholesterol (sample reduced by 5 %);.

c Model 3 adjusted for Model 2 plus baseline HDL-cholesterol and heart rate (sample reduced by 15 %).

d Model 4 adjusted for Model 1 plus baseline LDL-cholesterol and heart rate (sample reduced by 15 %).

A separate analysis of the predictive value of TyG and that of classical risk factors indicated a higher c-index of TyG (Harrell’s c-index: 0.55) than those of total cholesterol, LDL cholesterol, BMI, smoking, and renal function, similar to SUA and systolic BP, but lower than those of age and DM (Supplemental Table 4).

### 3.5 Subgroup analysis

Finally, subgroup analysis detected a significant predictive role of TyG on mortality in all but three subgroups (Table 3). In particular, the relationship was significantly more pronounced in participants younger than 70 years or with LDL cholesterol higher than 130 mg/dl. The predictive role of the TyG index disappeared in specific subgroups: participants with BP lower than 140 mmHg, those with DM, and individuals with renal dysfunction. However, this lack of predictive ability was not statistically different from the other groups (p for interaction >0.05). Additionally, in the subgroup analysis, other risk factors assessed -such as sex, smoking habits, SUA levels, and body weight- did not affect the predictive role of TyG regarding cardiovascular mortality risk.

**Table 3:** Subgroup analysis of the predictive role of the triglyceride-glucose (TyG) index on cardiovascular mortality.

	**Subgroups**	**N of participants**	**Cardiovascular Mortality**‡	**p-value**	**P for interaction**
			SHR (95 % CI)*		
Sex	Males	6101	1.53 (1.23–1.91)	<0.001	0.32
	Females	6174	1.78 (1.46–2.17)	<0.001	
Age	≥70 yrs	3525	1.28 (1.09–1.51)	0.003	0.001
	<70 yrs	8750	2.34 (1.71–3.20)	<0.001	
Type 2 Diabetes	Yes	1448	1.42 (0.92–2.21)	0.1	0.95
	No	10,827	1.40 (1.20–1.65)	<0.001	
Smokers	Yes	2774	1.62 (1.11–2.36)	0.01	0.95
	No	9501	1.64 (1.40–1.92)	<0.001	
LDL cholesterol†	≥130 mg/dl	5679	2.00 (1.57–2.55)	<0.001	0.04
	<130 mg/dl	4695	1.44 (1.17–1.77)	0.001	
Systolic BP	≥140 mmHg	10,067	1.67 (1.44–1.94)	<0.001	0.39
	< 140 mmHg	2208	1.19 (0.56–2.55)	0.6	
Renal function	eGFR ≤60 ml/min/1.73 m^2^	2017	1.45 (0.99–2.12)	0.05	0.43
	eGFR >60 ml/min/1.73 m^2^	10,258	1.71 (1.46–2.02)	<0.001	
High SUA	SUA≥5.6 mg/dl	4521	1.37 (1.08–1.72)	0.008	0.20
	SUA <5.6 mg/dl	7754	1.67 (1.38–2.02	<0.001	
Body weight	Normal	4210	1.76 (1.41–2.20)	<0.001	0.92
	Overweight	5543	1.66 (1.32–2.10)	<0.001	
	Obesity	2522	1.78 (1.19–2.66)	0.005	

BP: blood pressure, SUA: serum uric acid; eGFR: estimated glomerular filtration rate; SBH: sub-hazard ratio.

‡ TyG > 4.54 Units vs TyG ≤ 4.54 Units;.

⁎ Competing risk analysis;.

† sample reduced by 15 %.

## 4 Discussion

The results of this study show that TyG is a significant predictor of cardiovascular mortality in hypertensive people (Graphical Abstract). In particular, our analysis indicates that TyG is positively and linearly associated with cardiovascular mortality: TyG >4.54 was associated with a 67 % increased risk of cardiovascular mortality than lower values.

The predictive role of TyG was also independent of potential confounders, such as age, body weight, DM, BP levels, smoking habits, renal function, and lipid profile. In addition, it was greater than some classical risk factors (such as BMI, smoking, total cholesterol, LDL cholesterol, and renal function) and similar to those of BP and SUA. The results are substantially consistent with our previous analysis of a general population [12]; despite the detection of a different association shape (i.e. non-linear), a similar TyG threshold was still predictive of cardiovascular mortality.

Most previous studies that reported an association between the TyG index and cardiovascular risk or mortality were conducted in general or heterogeneous populations, often including individuals with established cardiovascular disease or pre-diabetes. Moreover, the majority of these investigations employed quartile-based analyses or focused primarily on all-cause mortality as the endpoint. In contrast, the present study specifically evaluates the prognostic value of the TyG index for cardiovascular mortality in a large, multicenter cohort of hypertensive individuals without a history of cardiovascular events, recruited from centers across Italy. Notably, this is the first study to identify and validate a clinically applicable TyG threshold (4.54 units) capable of stratifying cardiovascular mortality risk in this high-risk population. Additionally, the use of time-dependent receiver operating characteristic (ROC) analysis, competing risk models, and comprehensive subgroup analyses enhances the robustness and clinical relevance of our findings.

Subgroup analysis substantially confirmed the predictive role of TyG on cardiovascular mortality risk. Of note, the analysis shows that the predictive role of TyG on cardiovascular mortality was significantly more pronounced in participants younger than 70 years. This result is in agreement with the previous URRAH paper, in which the influence of metabolic patterns on cardiovascular risk was different with age [27]. The more pronounced predictive value of the TyG index for cardiovascular mortality observed in individuals younger than 70 years may reflect a greater pathophysiological significance of IR and its associated metabolic abnormalities in this age group. Younger hypertensive individuals with elevated TyG levels may exhibit more active or early-stage metabolic dysfunctions (e.g., impaired glucose metabolism, atherogenic dyslipidemia, and endothelial dysfunction) that disproportionately contribute to cardiovascular risk in the absence of overt clinical disease. In contrast, the prognostic impact of TyG in older individuals may be attenuated by the presence of competing risk factors, age-related vascular changes, or a survival effect, wherein older adults may represent a more resilient subset, less vulnerable to the adverse consequences of metabolic derangements. Further research is needed to elucidate the biological mechanisms underlying this interaction and to assess whether TyG-based risk stratification provides greater clinical value in younger hypertensive populations. Nonetheless, although the significant difference between subgroups, the predictive role of TyG was detected in both age subgroups. The predictive value of TyG on mortality seems also more pronounced in individuals with LDL cholesterol higher than 130 mg/dl. Probably, the interaction among IR, blood triglycerides and glucose with LDL cholesterol may interfere and attenuate the predictive power of TyG [28]. However, despite this difference, the TyG threshold significantly predicted cardiovascular mortality in both groups.

On the other hand, TyG was not associated with cardiovascular mortality risk in participants with systolic BP lower than 140 mmHg. Probably, the small sample size (*n* = 2243) and the low cardiovascular mortality rate (*n* = 30) may justify the neutral result in this group. Likewise, the findings indicated that the TyG index did not correlate with cardiovascular mortality risk in participants with renal dysfunction or DM. This lack of association could stem from the small sample sizes (*n* = 2017 for renal dysfunction and *n* = 1448 for DM) and the poorer baseline cardiovascular risk profiles observed in those who died, which included higher BP, older age, and lower renal function. These factors may interact with TyG and attenuate its predictive value in these specific populations. Nevertheless, in the analysis including the total sample, the adjustment for BP, DM, and renal function did not affect the predictive role of TyG on cardiovascular mortality. In addition, the subgroup analysis did not reveal other explored characteristics influencing the predictive role of TyG, namely, sex, body weight, smoking habit, and high SUA.

Although the TyG index was originally introduced over a decade ago, its adoption in cardiovascular epidemiology has substantially expanded in recent years. Several observational studies found a substantial trend of a positive relationship between TyG and cardiovascular disease and mortality [[11], [12], [13], [14]]. Moreover, some studies carried out in different settings suggest a direct association with cardiovascular risk factors [10,15,16,29,30]. However, published data of the predictive role of TyG on mortality in hypertensive people are few and heterogeneous [[17], [18], [19]]. One large prospective study including the NHANES hypertensive population (*n* = 8554, follow-up: 88 months) showed a non-linear relationship between TyG and all-cause or cardiovascular mortality [17]. In addition, the analyses indicated that values of the highest quartile of TyG were associated with a higher incidence of mortality than the lowest quartile. Notably, the different characteristics of the participants included in the study of Zhou et al. [17] (i.e. multiracial participants and also participants with cardiovascular disease) may justify the different shape of the association in the cardiovascular mortality risk found in our analysis. By contrast, another analysis of the NHANES database (*n* = 3614) found a significant association between TyG and all-cause mortality, but not with cardiovascular mortality by the quartiles evaluation [18].

Another large prospective study including an elderly hypertensive Chinese population (*n* = 8487) showed a significant association between TyG and first stroke events after only 1.7 years of follow-up [19]. The authors showed a linear trend across the quartiles of TyG on stroke events, but a potential non-linear association was not explored. Even in this context, no threshold was detected and participants with coronary heart disease were also included. Of note, in these papers [[17], [18], [19]] no cut-off was detected, therefore, the results of our study have advantages, because our threshold can be easily included in the cardiovascular risk stratification and applicable to the clinical practice. Moreover, in the literature, the mean values of the TyG index are reported using two ranges, due to a misunderstanding of the original formula [31]; although these two ranges do not generate a substantial difference in the relationship between the index and outcomes, this dissimilarity leads to a difficult comparison among studies results. Furthermore, some studies were performed to evaluate the predictive role of TyG on mortality in hypertensive individuals at high cardiovascular risk (i.e. with already explicit cardiovascular disease). Recent data from ONTARGET and TRASCEND, including 29,960 participants with chronic stable cardiovascular disease, indicated that higher TyG levels were associated with a modestly increased risk for incident cardiovascular events and LDL cholesterol levels largely attenuated the association of TyG with cardiovascular risk [32]. Also, a study on Chinese hypertensive people (*n* = 1467) with coronary heart disease, showed a positive association between TyG and cardiovascular mortality after one year of follow-up [33]. Whereas, another analysis on the NHANES database including hypertensive with coronary heart disease participants did not find any association between TyG and cardiovascular risk [34].

Our analysis indicated a worse metabolic and hemodynamic profile in those who had TyG values above the cut-off. These data may partially explain the higher risk of mortality in participants with greater than lower TyG values, given that TyG is an expression of the muscle and liver IR [35] and, in turn, well-known associated with several cardiovascular risk factors (e.g. BP, obesity, DM, etc.) [36]. Indeed, the significant association found between TyG and the cardio-metabolic risk factors (i.e., BP, anthropometric indices, lipid profile, SUA, and renal function) further supports the pathophysiological association among TyG-IR-cardiovascular risk [[36], [37], [38], [39]].

Finally, it should be noted that the definition of hypertension used in this study (systolic/diastolic BP ≥140/90 mmHg or current antihypertensive therapy) reflects the clinical guidelines at the time of data collection. While more recent recommendations suggest a lower threshold (130/80 mmHg) [40], our choice ensured consistency with national standards and previous epidemiological research. However, this may limit the generalizability of our findings to individuals classified as hypertensive under the newer criteria. Further prospective studies adopting updated definitions are needed to confirm the prognostic value of TyG across a broader hypertensive population.

### 4.1 Strengths and limitations

The results of this study are strengthened by: the soundness of the results; the large sample of hypertensive individuals from an Italian multicenter database; an extended follow-up period; careful reporting according to STROBE guidelines; detailed subgroup analyses; the identification of a TyG threshold for cardiovascular mortality in hypertensive individuals through time-dependent ROC analysis, for the first time; and performing a competing risk analysis and assessing the discriminatory power of the models and their relative goodness of fit. Therefore, the results of this study make this TyG threshold applicable to the routine clinical practice of cardiovascular prevention and risk stratification.

Nonetheless, our study has some limitations: 1) the observational design of the study does not permit the establishment of a causal relationship. 2) The enrollment of a homogeneous Caucasian population from Italy may limit the generalizability of the findings to other ethnic or geographic populations. Indeed, ethnic differences in metabolic profiles and cardiovascular risk may influence the performance of the TyG index, and healthcare system variations may affect its clinical applicability; hence, future validation in more diverse and international cohorts is warranted to confirm the external validity of the proposed threshold. 3) The potential influence of some unmeasured lifestyle-related variables (e.g., nutritional habits and physical activity) cannot be excluded. In particular, salt and potassium intake, both of which are closely linked to IR and cardiovascular risk [36,41], were not formally assessed in the present study. This is a relevant limitation, as excessive dietary sodium is known to increase salt sensitivity of BP, promote arterial stiffness, and enhance sympathetic nervous system activity, particularly in overweight or IR individuals [36,42]. Likewise, a low dietary intake of potassium has been associated with impaired insulin sensitivity and greater cardiovascular risk [41]. Future prospective investigations incorporating repeated TyG measurements and detailed nutritional profiling are warranted to clarify the potential mediating or modifying roles of these dietary exposures in the association between TyG and cardiovascular outcomes. 4) The analysis based on a single baseline TyG measurement may represent a limitation of the study. While the TyG index is a reproducible and simple surrogate marker of IR, its levels may fluctuate over time in response to changes in dietary intake, physical activity, or other metabolic influences. The absence of serial TyG measurements precludes an evaluation of the prognostic significance of TyG longitudinal trajectories. Although this limitation is inherent to the structure of the database, our findings nonetheless highlight the significant predictive value of a single baseline TyG determination for cardiovascular mortality. 5) The classification of cause of death, based on ICD-10 codes, which, despite their standardization and widespread use in epidemiological research, may introduce misclassification bias due to limited clinical specificity. However, their consistent use across all centers ensured uniformity in outcome assessment. Finally, 6) this study focused exclusively on cardiovascular mortality, a hard and specific endpoint with strong prognostic value and minimal risk of misclassification. However, the exclusion of nonfatal cardiovascular events, such as myocardial infarction, stroke, or heart failure, may limit a more comprehensive evaluation of the prognostic relevance of TyG across the full spectrum of cardiovascular disease. Future analyses are planned to explore the association between TyG and nonfatal outcomes, which may further enhance the clinical utility of this marker in risk stratification.

## 5 Conclusions

The main findings of this study indicate that TyG is positively and linearly associated with cardiovascular mortality in a large sample of hypertensive individuals from a Caucasian general population. Notably, this study for the first time detected a TyG threshold of 4.54, which is able to identify the additional cardiovascular risk in individuals who are already considered at high risk, independently of other potential confounding factors. Moreover, the predictive value of TyG seems to be more pronounced in participants younger than 70 years old, or those with elevated LDL cholesterol levels. These results suggest that TyG may serve as a low-cost and simple non-invasive marker for cardiovascular risk stratification in hypertensive people. Nonetheless, further studies are needed to support our results.

## Ethics approval and consent to participate

The URRAH study was performed according to the Declaration of Helsinki for Human Research (41st World Medical Assembly, 1990). Approval was sought from the Ethics Committee of the 7 coordinating center at the Division of Internal Medicine of the University of Bologna (no. 77/2018/Oss/AOUBo). Informed consent was obtained from all subjects included in the study.

## Use of AI and AI-assisted technologies statement

During the preparation of this work, the authors did not use any automated authoring tools or services.

## CRediT authorship contribution statement

**Lanfranco D’Elia:** Writing – original draft, Visualization, Software, Methodology, Investigation, Formal analysis, Conceptualization. **Ferruccio Galletti:** Writing – original draft, Validation, Supervision, Methodology, Investigation, Formal analysis, Conceptualization. **Masulli Maria:** Writing – review & editing, Investigation. **Agostino Virdis:** Writing – review & editing, Resources, Project administration, Investigation, Funding acquisition, Conceptualization. **Edoardo Casiglia:** Writing – review & editing, Visualization, Software, Resources, Project administration, Investigation, Funding acquisition, Data curation. **Valerie Tikhonoff:** Writing – review & editing, Visualization, Software, Resources, Project administration, Investigation, Funding acquisition, Data curation. **Fabio Angeli:** Writing – review & editing, Investigation. **Carlo Maria Barbagallo:** Writing – review & editing, Investigation. **Michele Bombelli:** Writing – review & editing, Investigation. **Federica Cappelli:** Writing – review & editing, Investigation. **Rosario Cianci:** Writing – review & editing, Investigation. **Michele Ciccarelli:** Writing – review & editing, Investigation. **Arrigo F G Cicero:** Writing – review & editing, Investigation. **Massimo Cirillo:** Writing – review & editing, Investigation. **Pietro Cirillo:** Writing – review & editing, Investigation. **Giovambattista Desideri:** Writing – review & editing, Investigation. **Claudio Ferri:** Writing – review & editing, Investigation. **Loreto Gesualdo:** Writing – review & editing, Investigation. **Cristina Giannattasio:** Writing – review & editing, Investigation. **Guido Grassi:** Writing – review & editing, Investigation. **Guido Iaccarino:** Writing – review & editing, Investigation. **Luciano Lippa:** Writing – review & editing, Investigation. **Francesca Mallamaci:** Writing – review & editing, Investigation. **Alessandro Maloberti:** Writing – review & editing, Investigation. **Stefano Masi:** Writing – review & editing, Investigation. **Alberto Mazza:** Writing – review & editing, Investigation, Conceptualization. **Alessandro Mengozzi:** Writing – review & editing, Investigation. **Maria Lorenza Muiesan:** Writing – review & editing, Investigation. **Pietro Nazzaro:** Writing – review & editing, Investigation. **Paolo Palatini:** Writing – review & editing, Investigation. **Gianfranco Parati:** Writing – review & editing, Investigation. **Roberto Pontremoli:** Writing – review & editing, Investigation. **Fosca Quarti-Trevano:** Writing – review & editing, Investigation. **Marcello Rattazzi:** Writing – review & editing, Investigation. **Gianpaolo Reboldi:** Writing – review & editing, Investigation. **Giulia Rivasi:** Writing – review & editing, Investigation. **Elisa Russo:** Writing – review & editing, Investigation. **Massimo Salvetti:** Writing – review & editing, Investigation. **Giuliano Tocci:** Writing – review & editing, Investigation. **Andrea Ungar:** Writing – review & editing, Investigation. **Paolo Verdecchia:** Writing – review & editing, Investigation. **Francesca Viazzi:** Writing – review & editing, Investigation. **Massimo Volpe:** Writing – review & editing, Investigation. **Claudio Borghi:** Writing – review & editing, Visualization, Resources, Project administration, Investigation, Funding acquisition, Conceptualization.

## Declaration of competing interest

The authors declare the following financial interests/personal relationships which may be considered as potential competing interests:

URRAH study group reports administrative support was provided by Fondazione of the Italian Society of Hypertension. Claudio Borghi reports a relationship with Menarini Corporate, Novartis Pharma, Alfasigma, Laboratoires Servier, Grunenthal, Takeda, Astellas, Teijin, Berlin Chemie, Sanofi that includes: consulting or advisory, funding grants, and speaking and lecture fees. The remaining authors have no disclosures to report.

## Appendix Supplementary materials

Supplemental Table 1. STROBE Statement—Checklist of items that should be included in reports of cohort studies. Supplemental Table 2. Characteristics of excluded participants and comparison with those included in the final analysis. Supplemental Table 3. Baseline characteristics of the study participants stratified by cardiovascular mortality. Supplemental Table 4. C-index of the triglyceride-glucose index and classical risk factors. Image, application 1
